# A Meta-Analysis of the Effects of Interaction on Value Co-Creation in Online Collaborative Innovation Communities Based on the Service Ecosystem Framework

**DOI:** 10.3390/bs14121177

**Published:** 2024-12-09

**Authors:** Chunzhen Wang, Xin Zhao, Jianzhong Hong

**Affiliations:** 1 Key Laboratory of Adolescent Cyberpsychology and Behavior (CCNU), Ministry of Education, Wuhan 430079, China; wangchunzhen@mails.ccnu.edu.cn; 2 Key Laboratory of Human Development and Mental Health of Hubei Province, School of Psychology, Central China Normal University, Wuhan 430079, China; 3 Manchester Institute of Education, University of Manchester, Manchester M13 9PL, UK

**Keywords:** interaction, value co-creation, online collaborative innovation communities, service ecosystem framework, meta-analysis

## Abstract

Interaction is typically at the core of the value co-creation process through operant resource exchange in online collaborative innovation communities (OCICs). While some studies emphasize the facilitating effect of interaction on value co-creation, others have drawn opposite conclusions, such as more peer interaction leads to less idea generation. Thus, the purpose of this paper is to utilize the service ecosystem framework to clarify the overall relationship between interaction and value co-creation and to explore the moderating factors that may have contributed to the divergence and inconsistency of previous studies. We conducted a meta-analysis of 65 effect sizes obtained from 63 articles with a cumulative sample size of 25,185 between 2004 and 2023, using a random effects model. The results indicate that interaction has a significantly positive impact on user value co-creation within OCICs (*r* = 0.453, 95%CI [0.405, 0.499]), and the heterogeneity among studies was significant (*Q* = 1409.29, *p* < 0.001). The strength of this correlation was moderated by the types of interaction (human–computer or human–human interactions), the types of OCICs (business-sponsored or socially constructed online communities), and the number of involved OCICs (one or multiple online communities), but not by the cultural background. These findings support the service ecosystem perspective rather than resource scarcity theory by resolving the mixed findings regarding the relationship between interaction and user value co-creation. Furthermore, this study systematically examined the contingent factors separately across three levels, micro (types of actor interactions), meso (types and number of OCICs), and macro (cultural background), combining the whole and the part insights, and empirically integrating service ecosystems as the foundational paradigm and unit of analysis for value co-creation research for the first time. This research contributes to theoretical frameworks in service ecosystems and offers actionable insights for management practices in business and marketing.

## 1. Introduction

*By offering mutual value—through the community you create […] can create a powerful brand that stands the test of time and becomes a force to be reckoned with in its niche […] creating mutual value is best achieved through strong internal collaborations…*

—Kate Vitasek, Forbes, 8 November 2023

In the digital economy era, digitalized technological advances in artificial intelligence and machine learning have significantly accelerated the development of the new organizational form of the technology-enabled online community globally. These online platforms are increasingly empowering Internet users to access, share, and create diverse information and knowledge [1]. One of the most noteworthy is the online collaborative innovation communities (OCICs), which serve as highly interconnected and interactive platforms that actively facilitate user engagement in value co-creation processes [2]. A growing number of organizations are leveraging OCICs (e.g., transaction-based online communities like Starbucks’ MyStarbucksIdea, online social Q&A communities like Zhihu and online health communities like Haodaf.com) to collaborate with users or customers to access external innovation resources and integrate them into the internal innovation process of the business or non-profit organization to achieve greater business performance and social benefits [3].

Value co-creation in the OCICs refers to all actors in the OCIC platform ecosystem exchange and integrate resources and engages in mutually beneficial interactive cooperation processes to create perceived value-in-use and value [4,5]; activities like knowledge sharing [6] and generating new ideas and solutions [7] are core events. This emerging phenomenon of value creation has profound implications for both organizational management strategies and broader economic development models. From a multi-actor perspective, value co-creators are all actors within an OCIC that collaborate and interact as well as contribute resources, including not only human actors (stakeholders, e.g., users, customers, platform moderators, suppliers, and business partners [2]), but also nonhuman actors like interactive platforms and artifacts [8,9]. Crucially, without ongoing dynamic interaction between human and nonhuman actors to achieve win-win situations in OCICs, value co-creation is difficult to occur [10,11].

Online community interaction is the mutual or reciprocal action or influence of multiple relevant actors taking place in online communities, primarily involving the process of exchanging and integrating operant resources—intangible resources such as information, knowledge, and skills [4,12,13]. Since the realization of value co-creation is possible in the context of interaction, actor-to-actor interaction represents the crux of OCICs’ effectiveness and prosperity [4]. Consequently, scholars have examined the relationship between actor interaction and value co-creation, both theoretically and empirically. Most of the related studies concluded that interaction among actors positively influences value co-creation based on the traditional service-dominant (S-D) logic [2,14]. For example, through the lens of reciprocity norms, it is evident that sharing valuable information and facilitating positive interactions enhance relationship building and interest in each other’s ideas among community members, which in turn increases their willingness to engage in value co-creation in OCICs [15]. However, interaction in the online community does not always yield positive outcomes. On the premise that attention is a scarce resource, other studies have found an inverted U-shaped [16] or even negative [17] relationship between interactions and value co-creation. Specifically, since individuals have limited attentional resources to process information [18], users who interact heavily in OCICs will experience information overload, which depletes users’ cognitive resources to absorb useful knowledge and specialize in innovation. Furthermore, value co-destruction in OCICs may even be triggered when unpleasant and inappropriate interactions hinder users from integrating resources in a reciprocal and mutually beneficial way [19,20]. While dyadic interactions have been extensively studied, the multi-level dynamics in OCICs remain underexplored, warranting further investigation.

To sum up, we found that there is still no consensus on the relationship between actors’ interaction and value co-creation in OCICs since the direction and magnitude of the correlation coefficients differ significantly [17,21]. Moreover, few previous studies have examined simultaneously how interactions between human and nonhuman actors affect value co-creation in OCICs in the digital background, and there is a need for further consideration of the different impacts of multiple interaction types on value co-creation. As interactions occurring in digital platform ecosystems like OCICs are bridged by platforms, the interactive platforms are both service providers and mediated actors [9]. Drawing on the ecological systems theory introduced by Moore (1996) in the field of management [22] and recent developments in S-D logic [13], there is growing recognition of the need to examine triad interactions between parts and wholes (i.e., actors and interactive platforms) across meso levels beyond micro-level interactions among actors, and thus beyond traditional micro-level analyses. These gaps make it difficult for community administrators to develop and design a vivid and effective interaction environment to cultivate the norm of reciprocity among users, based on the characteristics of different OCICs and types of interactions. By addressing these inconsistencies, it will be possible to increase the understanding of multi-actor interactions in digital ecosystems and better facilitate user engagement in value co-creation.

To address the inconsistencies in prior research and further explore the factors influencing interaction and value co-creation across micro, meso, and macro levels from the multi-actor perspective, this study seeks to answer the following two research questions based on service ecosystem theory and using a meta-analysis approach:

RQ1. What are the relationships between interaction and value co-creation in OCICs?

RQ2. To what extent are the above relationships moderated by underlying factors at different levels (macro, meso, and micro) within the interactive platform ecosystem, and how do these moderators affect the main effects?

## 2. Literature Review and Research Hypotheses

### 2.1. Structure and Connotation of User Value Co-Creation

With the growth of the digital economy and virtual communities, the conceptualization of value co-creation has evolved with the guidance of service ecosystem theory, which is seen as the process of creating value for the online community through social interactions and network relationships among all actors in the platform ecosystem [23,24,25]. Previous research has argued that value co-creation involves diverse self-generated activities [2]. Concerning Akman et al.’s (2019) summary of value co-creation activities and other relevant literature, user value co-creation in OCICs can be primarily categorized into two types of behaviors: in-role participation behavior that consists mainly of information seeking, information sharing, and responsible behavior [2,26], and extra-role citizenship behavior, which is voluntary and capable of contributing extraordinary value to OCICs, with feedback and advocacy being two of the common behaviors [27]. Therefore, the above five typical representations of value co-creation behavior are incorporated as outcome variables in our meta-analysis. Furthermore, internet-mediated value co-creation occurs sometimes through crowdsourcing, in which users beyond designated agents collaboratively seek innovative solutions for new products and evaluate others’ ideas to make contributions [16]. Thus, creative and new idea generation is integrated into this study as a successful value co-creation behavior generated in the crowdsourcing process. Additionally, our meta-analysis also includes terminology that is synonymous with value co-creation and its key indicators, such as co-innovation and user innovation [28].

### 2.2. Service Ecosystem Framework

Originally, numerous studies used S-D logic as a theoretical foundation to explore how customer–firm dyadic interactions affect value co-creation for business competitive advantage at the micro level [8]. However, with the advancement of digital technologies, scholars have changed their focus from “dyad interaction” to “multi-actor network interaction”. This shift is framed through the service ecosystems lens, which has become a dominant research paradigm for exploring value co-creation in interactive platforms supported by Internet technologies [9]. Service ecosystems, integrating Moore’s ecological systems theory and S-D logic, are defined as relatively self-contained, self-adjusting systems of multiple actors connected through mutual value creation, where actors interact through resource exchange and integration to achieve value co-creation [29]. The service ecosystem framework encompasses three levels: micro, meso, and macro. This emphasizes the importance of considering the dynamic interplay between different subsystems across levels. The micro-context frames a reciprocal dyadic interaction between actors, whereby both actor parties use their resources and competencies to initiate service-for-service exchange and resource integration, which is an instrumental exchange process enabling the co-creation of value [30]. The meso level, as an addition of mediated actors beyond traditional actor-to-actor dyadic interactions, exhibits service exchange linkages between direct stakeholders [30]. The macro level in the service ecosystem represents the influence of the larger external social and cultural environment acting on human actors. It ultimately constitutes a loosely interacting ecosystem by including more marginal actors and organizations [30].

### 2.3. The Relationship Between Interaction and User Value Co-Creation

From the micro level of the service ecosystem framework, value co-creation needs to be built through actors’ interactions in OCICs. Increased interaction gives rise to interdependence among actors for the exchange, transformation, and integration of operant resources. Moreover, relevant studies have demonstrated that actors share and exchange important information and knowledge through reciprocal interactions. For users, it cognitively increases their perceptions of fairness and transparency [31], while it interpersonally strengthens the social bonds and trust of community members [6]. For firms, it optimizes the company’s innovation capabilities and service quality by learning about users’ needs [32]. Collectively, these dynamics exert a positive influence on value co-creation within OCIC platform ecosystems. To sum up, we hypothesize the following:

**H1.**  *Interaction between actors in OCICs has a significant positive effect on user value co-creation behavior.*

### 2.4. Moderating Effects

Since online community platforms are complex systems of activities, there remains a notable gap in the literature regarding how various components of the ecosystem (multiple actors, OCIC platform organization, and external cultural environment) affect the relationship between interactions and value co-creation from a holistic perspective across levels. Therefore, our study tries to address this gap by examining the effects of the moderators from a dynamic perspective across the micro, meso, and macro levels, respectively, based on the service ecosystem framework.

#### 2.4.1. Micro-Level Dynamics: Types of Interaction Among Actors

In the service ecosystem framework, the role of interaction types at the micro level should not be overlooked. Given that value is co-created by multiple actors [13], it is necessary to examine the impact of different types of actor’s interactions on the value co-creation within OCICs. In OCICs, which are embedded in digital and intelligence technologies, actors include ‘hybrid collectives’ of human and nonhuman entities (such as artifacts and processes) [9]. Correspondingly, interactions occurring between multiple actors comprise both human–human interaction (HHI) and human–computer interaction (HCI).

HHI refers to the exchange of resources and communication between human actors beyond temporal and geographical constraints by sharing experiences, ideas, and information through the Internet as a medium [33]. A prominent form of HHI within OCICs is user–user interaction, which involves the exchange and interaction of comments, ideas, and emotions among users in OCICs [6,34]. With increased user–user interactions, there is a strengthening of perceived identity similarity [35] as well as the acquisition of intangible and operant resources from peers [4]. On the principle of reciprocal norms, users are incentivized to contribute more novel and high-quality ideas to the value co-creation process in OCICs [36]. Another significant form of HHI is user–firm agent interaction. Firm agents are typically mediated representatives of commercial organizations who are key stakeholders in OCICs other than the users [37]. User–firm agent interaction is essentially a formal interaction, occurring within firm-created OCICs that are primarily dominated or controlled by the companies to maintain a competitive advantage [38]. While companies can encourage users to continuously contribute valuable ideas through diverse interaction strategies [39], users may perceive these interactions as more obligatory, leading to lower engagement in value co-creation compared to the more informal nature of user–user interactions.

HCI is the most prevalent type of nonhuman actor–human actor interaction within OCICs. It refers to the process by which users interact with OC toolkits, aided by invisible algorithms and protocols, to conduct operations such as browsing, searching, and editing information [33]. Platform technological features like synchronicity and two-way communication in HCI processes can enhance user’ interactive experiences, fostering their willingness to co-create value [21]. Yet, from the perspective of technology acceptance [40], the presence of algorithmic “black boxes” at the technological level may diminish user agency, potentially undermining the value co-creation. In addition, HCI in the computer-mediated environment reflects the responsiveness of OCs and emphasizes a platform’s usability and channel capacity [41]. Therefore, HCI within different types of OCICs may affect the user’s interactive experience, and further consideration needs to be given to the impact of the type of OCICs.

More importantly, in the complex and dynamic environments of OCIC-based interactive platforms, multiple-actor interactions often occur simultaneously. As the variety of interactions within an OCIC increases, the platform becomes more dynamic, which can further motivate users to co-create value [14]. Thus, we hypothesize the following:

**H2.**  *The types of interaction among actors significantly moderate the relationship between interaction and user value co-creation. Specifically, user–user interactions have stronger effects compared to user–firm agent interactions and HCI. In addition, the greater the number of interaction types involved in OCICs, the stronger the correlation between interaction and value co-creation.*

#### 2.4.2. Meso-Level Expansion: Types of Online Collaborative Innovation Communities

Beyond the types of interactions, the structure and organization of OCICs play a crucial role in shaping the dynamics of value co-creation. Within the service ecosystem framework, the OCICs types are important at the meso level, because as a type of digitalized interactive platform, OCICs are active mediators that provide virtual interfaces for actors’ value co-creation [9]. Therefore, the affecting factors of value co-creation in OCICs should be examined across both micro and meso levels of the platform ecosystems, situated within the broader context of interactive system environments where actors are embedded. Altogether, we propose that different types of OCICs may influence the relationship between interaction and user value co-creation.

Drawing on established typologies of virtual communities [42,43,44], OCICs oriented towards business innovation can be categorized into three types: generalized transaction-based communities, crowdsourcing communities, and brand communities embedded in social media. Within these OCICs, members primarily engage in idea submission as the core activity [45]. Additionally, two socially constructed online communities dedicated solely to interest and information exchange are also classified as OCICs, including interest-based and relational online communities [46]. Although these particular OCICs may not be explicitly designed to stimulate users’ creative behaviors, collaborative innovations frequently emerge organically through actor-to-actor interactions [2]. The description and operationalized definition of specific types of OCICs included in our meta-analysis are listed in Table 1, from which the purpose, characteristics, and examples of different OCICs can be clearly accessed, as well as the number of different types of OCICs included in the meta-analysis.

The moderating effects of OCIC types may be driven by differences in the actor’s strength of social bonds and intentionality of gathering [47]. Specifically, in generalized transaction-based online communities, as firms use OCICs to connect with users and gather valuable information, the platform’s administrators are responsible for monitoring user speech and influencing public opinion [48]. Consequently, this community structure often restricts flexibility in actor-to-actor interactions and leads to relatively weaker social bonds and interdependencies among actors [49], thereby reducing users’ willingness to actively co-create value. In contrast, in crowdsourcing communities, users have more isolated memberships and less intentionality of gathering and collaborating, as they primarily engage in one-time, short-term crowdsourcing contests, submitting ideas separately [50]. The limited connectivity within crowdsourcing communities tends to diminish the positive influence of interactions on users’ value co-creation. Further, brand communities embedded in social media like Facebook and Sino Microblogs have emerged as pivotal mediators for companies to connect the community triangle relationship between brands and customers and other customers [41]. Since members are mainly brand fans or supporters who share common interests and preferences, the strength of emotional bonds and personal connections among users as well as the sense of community identity and belonging are stronger [51]. Thus, users are more willing to remain within brand communities for ongoing interaction, participating actively in brand-driven value co-creation efforts. In comparison, online interest-based and relational communities represent highly connected social networks built tightly around social or professional relationships between members, free from commercial influence or firm control [46]. While these online communities may lack the broad accessibility and convenience of online communities embedded in social media, they are characterized by greater member cohesion, stability, and stronger emotional bonds [52]. Further, users in online relational communities share more similar concerns and experiences, with higher levels of social support and willingness to mutual assistance, which drives more intentional and proactive knowledge sharing [53]. Hence, the following hypothesis is proposed:

**H3.**  *The types of online collaborative innovation communities significantly moderate the relationship between interaction and value co-creation. Specifically, brand communities embedded in social media have the most significant effect, followed by two types of socially constructed online communities where online relational communities have a relatively stronger effect and crowdsourcing communities have the weakest effect when compared to other types of OCICs.*

#### 2.4.3. Meso-Level Expansion: The Number of Involved Online Collaborative Innovation Communities

Besides the type of OCICs, the number of OCICs included can likewise affect the relationship between interaction and value co-creation. Further, within the service ecosystem framework, the number of involved OCICs also plays an essential role at the meso level because there are multiple connected individual-to-system-to-individual system environments of platformed interaction at this level [9]. Through the configuration of multiple related interactive platforms, an “all win more” approach to value co-creation becomes achievable. Thus, actors’ interactions across a variety of interactive platforms can significantly enhance users’ experiences of value co-creation. Compared to studies considering only a single OCIC, the inclusion of multiple OCICs simultaneously implies a diversification of interactive platforms and an expansion of communication channels, which contributes to the development of relational networks among actors and increases the frequency and quantity of actors’ interactions [54]. In addition, studies focused on multiple OCICs take into account differences between various types of communities, with group heterogeneity and non-typicality favoring the generation of a diversity of innovative ideas [55]. Therefore, we propose the following hypothesis:

**H4.**  *The number of involved OCICs moderates the relationship between interaction and value co-creation. Specifically, there will be a stronger positive correlation between interaction and value co-creation in a study that includes multiple OCICs together compared to a study that includes only one OCIC.*

#### 2.4.4. Macro-Level Impacts: Cultural Background

Other than the type of interaction and the structure and number of OCICs, the influence of the cultural environment on value co-creation is equally worthy of attention. Within the service ecosystem framework, cultural background plays an essential role at the macro level because the individual’s cultural background (i.e., collectivism and individualism) are important determinants in shaping interaction dynamics and value co-creation. In the background of collectivist-oriented Eastern cultures, individuals are more willing to exchange resources within close-knit circles [56]. While OCICs are characterized by anonymity and weaker emotional ties among members [57], it is difficult to promote members’ value co-creation within Eastern cultural backgrounds through limited interactions in OCICs. Nevertheless, membership identity is not overemphasized in the background of individualist-oriented Western cultures [58]; the trust and rapport that anonymous members develop through interaction can effectively encourage the generation of creative ideas [59]. Thus, we propose the following hypothesis:

**H5.**  *The cultural background of OCIC users moderates the relationship between interaction and value co-creation. The positive impact of interaction on value co-creation is more pronounced among users from individualist Western cultures than those from collectivist Eastern cultures.*

In summary, firstly, previous studies show limited exploration of multi-actor interactions due to over-reliance on dyadic interactions. This should be further discussed as there are conflicting results on the impact of user–firm interaction and user–user interaction on the user’s value co-creation. Meanwhile, more attention should be paid to the impact of human–computer interaction in digital contexts. Second, previous studies show a narrow focus on a single OCIC, ignoring the important role of focusing on multiple OCICs at the same time in order to expand communication channels for value co-creation. In addition, the role of different types of OCICs with unique characteristics has been overlooked, and understanding how the types of OCICs affect the relationship between interaction and value co-creation can also provide further design guidance for online platform managers. Moreover, previous studies have been limited in exploring the moderating role of different cultural contexts. To summarize, as can be seen in Table 2, we aimed to overcome the limitations of previous studies through the above five hypotheses.

**Figure 1:**
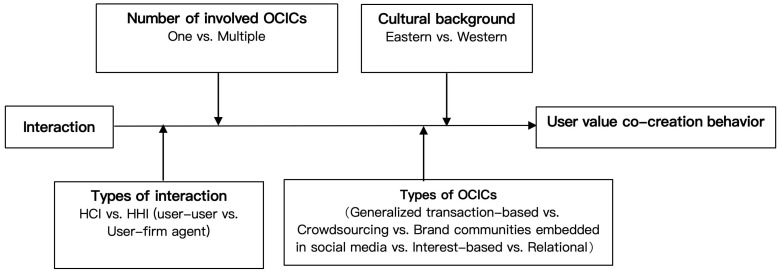
A research model of the relationship between interaction and value co-creation.

The overall conceptual framework model of this study is presented in Figure 1. It mainly presents the relationship between interaction and user value co-creation with the four moderating variables that we have hypothesized (i.e., types of interaction, types of OCICs, number of involved OCICs, and cultural background).

## 3. Methodology

### 3.1. Literature Search

**Figure 2:**
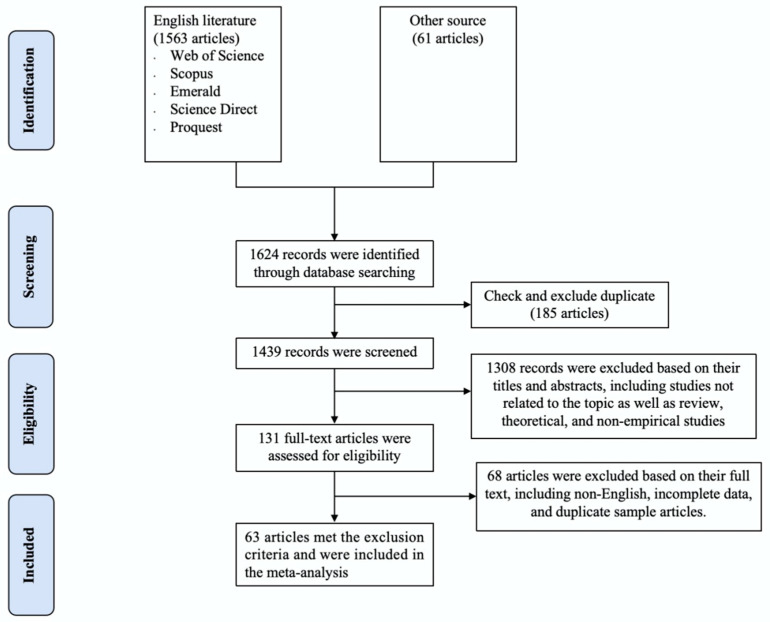
PRISMA study screening process diagram.

To identify relevant studies of the impact of interaction on value co-creation in OCICs, we retrieved references first from Web of Science, Scopus, Emerald, Science Direct, and Proquest. The searched keywords were all possible combinations of terms reflecting interaction, value co-creation, and online communities, specifically, the following combinations were used to search the database: (“interaction” OR “interactivity” OR “interactive activity”) AND (“value co-creation” OR “new idea co-creation” OR “creative idea generation” OR “co-creat value” OR “co-innovation” OR “crowdsourcing”) AND (“online community” OR “virtual community” OR “online platform” OR “online collaborative innovation community” OR “crowdsourcing community” OR “social network” OR “SNS” OR “Web 2.0 platform”). A total of 1563 articles were retrieved from the preliminary search database. Second, to ensure the comprehensiveness and representativeness of the meta-analysis samples and avoid omissions, we manually screened prestigious journals that are likely to publish research on the topic of online communities, such as the International Journal of Information Management, Journal of Computer-Mediated Communication, MIS Quality, Telematics and Informatics. In addition, we supplemented the literature by backtracking key references. A total of 61 studies were obtained. Through a multi-channel literature search, 1624 articles between 2004 and 2023 were identified. The literature screening process diagram is shown in Figure 2. It presents the process of search and selection protocols for identifying papers included in the meta-analysis; as can be seen from the figure, the process consists of four steps, i.e., identification, screening, eligibility, and inclusion.

### 3.2. Literature Inclusion Criteria

The retrieved studies were selected to be included in the meta-analysis if they met the following inclusion criteria:

(1) We first removed duplicate literature downloaded more than once from the same or different databases from the retrieved literature list to avoid methodological inadequacies [60]. (2) Since we examined the relationship between interaction and value co-creation in online collaborative innovation communities, the research background had to be the online community, and literature related to value co-creation in offline contexts was excluded. (3) As meta-analysis requires statistical data such as correlation coefficients between variables and sample sizes to be analyzed and tested, the studies had to be empirical research on the relationship between interaction and user value co-creation, excluding theoretical research, literature reviews, qualitative research and meta-analyses that did not contain correlation coefficients or could not be converted into correlation coefficients. (4) The literature had to include the sample size and the r-value of the zero-order correlation coefficient between interaction and value co-creation, or included other effect size indicators that can be converted into *r*-values (e.g., t, *F*, *χ*^2^) to be examined using meta-analysis. (5) The survey data involved in the literature were not used repeatedly. If the dissertations and conference papers were officially published in journals after revision, data from journal papers were preferred. (6) To avoid duplication of samples, multiple studies using the same sample were considered as one single study, and multiple independent samples within a study were treated as different observations. As shown in Figure 2, the final meta-analysis was based on 65 studies from 63 articles (one article conducted three empirical studies) involving 25,185 participants.

### 3.3. Variable Coding

This paper followed the steps recommended by Lipsey and Wilson (2001) to encode the 65 studies included in the meta-analysis [61]. We coded the included literature mainly using two parts. One is the sample characteristic description, such as basic literature information (e.g., author name and publication year) and hypothesized moderators, and the other is the statistical description of effect size. Variables were coded based on their definition and meaning. In this paper, two authors conducted the coding work independently after forming the coding sheet. The results showed that only individual data deviation exists in the two coding results; the intercoder agreement was 96.8%, indicating a high degree of consistency [62]. The two authors resolved a few discrepancies by discussion. In cases of discrepancies, the authors discussed solutions by referring to predefined coding guidelines. The basic information of 63 articles included in the meta-analysis is shown in the Supplementary Materials (for detailed data, please see Supplementary Table S1).

### 3.4. Effect Size

The effect size metric selected for the meta-analysis was the zero-order correlation coefficient, measuring the association between interaction and value co-creation in online communities. The effect sizes were coded such that a positive sign indicates a positive behavior change (i.e., an increase in value co-creation behavior). For papers that reported other statistics (e.g., *p*-value, *t*-value, *F*-value, *χ*^2^), we converted those statistical metrics to r following guidelines for meta-analysis [61,63]. If multiple variable indicators were measured simultaneously in the same study, we coped by selecting the value corresponding to the most comparable measured variable as the effect size. When comparisons were not possible, the weighted average of multiple measurement variables was included as the effect size of the meta-analysis.

### 3.5. Moderators

Some key research features were coded in the present meta-analysis to better understand what factors might have contributed to the inconsistent findings of previous studies on the relationship between interaction and value co-creation in OCICs. The moderators addressed in this study included categorical moderators: interaction type, OCIC type, number of involved OCICs, and cultural background. When the variables were categorical moderators, subgroup analysis was used to test whether the results were significant. In addition, the operationalized definitions and selection criteria of the moderators are summarized in Table 3.

Types of interaction: We coded the interaction type between different actors into three categories: human–human interaction, including user–user and user–firm agent interaction, and human–computer interaction. In the literature review above, the types of interaction and their functions were defined and discussed in detail.

Types of online collaborative innovation communities: OCIC types were categorized into five levels: interest-based communities, relational communities, generalized transaction-based online communities, crowdsourcing communities, and brand communities embedded in social media. The literature review above discussed the details of definitions and main characteristics of different OCICs.

Number of involved online collaborative innovation communities: This moderating variable represented the number of involved OCICs examined in each study. We coded it into two categories: one OCIC and multiple OCICs.

Cultural background: The moderator was the different cultural backgrounds to which the study sample belonged. We grouped the studies into two types of cultural backgrounds: Eastern (e.g., China) and Western (e.g., USA and UK).

### 3.6. Meta-Analysis Procedures

We used the correlation coefficient r as the effect size metric, and all analyses were conducted using Comprehensive Meta-Analysis Version 3.0 (CMA; [63]). First, this study used the approach recommended by Hedges and Vevea (1998) to calculate Fisher’s Z and the combination of effect sizes [64]. Second, we tested the heterogeneity of effect size and confirmed the model selection (i.e., whether to use a fixed-effects model or a random-effects model). The current study used the *Q*-statistic test and the relative statistics *I*-squared (*I*^2^) and *τ*-squared (*τ*^2^) to conduct the heterogeneity analysis. In particular, the *Q*-statistic measures the variability among effect sizes, while *I*^2^ indicates the proportion of variation due to real differences rather than random error. *τ*^2^ can be used to calculate the weights for each study, which measures the variance of the true effect sizes. Next, we used the Funnel plot test, Egger’s regression coefficient, Classica fail–safe *N*, and the trim-and-fill method to detect publication bias. Finally, to explain the heterogeneity in the relationship between interaction and value co-creation, the effect size of each variable relationship was calculated to test the moderating effects of interaction type, OCIC type, number of involved OCICs, and cultural background. Meanwhile, the results of the heterogeneity test, main effect test, and moderating effect test were reported.

## 4. Research Results

### 4.1. Heterogeneity Test

The purpose of the heterogeneity test is to examine variation among effect sizes and define appropriate moderators to capture and demonstrate such variation [65]. If there is no heterogeneity across the effect sizes, a fixed-effects model is used in the main effects analysis; otherwise, a random-effects model is used [64]. Table 4 is primarily a presentation of the results of the heterogeneity test and the results of the heterogeneity test indicated that the *Q* values were statistically significant (*Q* = 1409.29, *p* < 0.001), which is greater than the chi-square critical value of 83.675 corresponding to the degree of freedom of 64 at the 95% confidence interval level, indicating that there was heterogeneity among the effect sizes and allowing further potential moderating effect tests. In addition, *I*^2^ = 95.459%, which exceeds the 75% criterion proposed by Huedo-Medina et al. (2006), suggesting that the results were highly heterogeneous and therefore, a random-effects model was chosen over a fixed-effects model in this study [66]. It also indicated that about 95.459% of the observed variance was caused by true differences in effect sizes and only 4.541% of the variance was derived from random error. The analysis results were more stable. Furthermore, *τ*^2^ = 0.055, indicating that 5.5% was available to calculate weights across the studies, and the results of the heterogeneity test for the overall effect were statistically significant. Consequently, it is appropriate to use a random-effects model in analyzing the main effects of interaction and value co-creation relationships in OCICs.

### 4.2. Publication Bias

Beyond using the heterogeneity test to select the appropriate model to define the moderating variables, we conducted a publication bias test to reveal the effects of nonsignificant and unavailable studies [64]. As statistically significant studies are more likely to be published than statistically insignificant studies, it is necessary to test for publication bias in meta-analyses that include more published studies. Publication bias refers to the fact that published studies may not be sufficiently representative of the overall study, causing the effect sizes of the meta-analysis to deviate from the true values [67]. We assessed and minimized publication bias through the following methods. First, we tackled the problem of publication bias to the extent possible through more comprehensive database searches. The present study included not only published journal papers as well as conference papers and dissertations that were not formally published in the literature screening process, controlling to some extent the interference of publication bias on the study results [68]. Moreover, to ensure the robustness and reliability of the study findings, we assessed publication bias by additional multiple methods, i.e., Funnel plots, Egger’s regression coefficient, fail-safe *N*, and trim-and-fill method [69].

**Figure 3:**
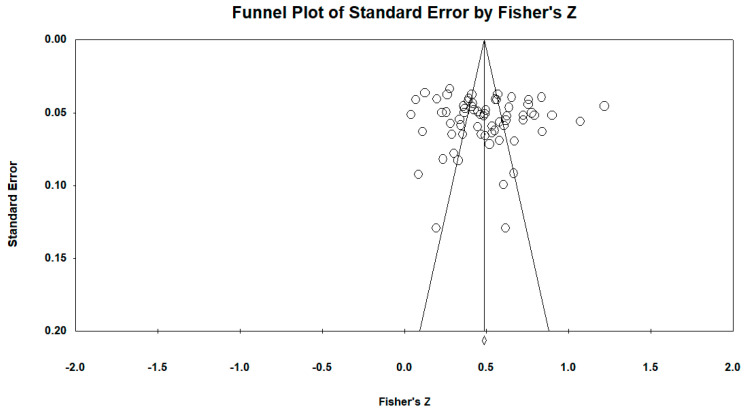
Funnel plot of effect sizes.

As shown in Figure 3, the effect sizes included in the meta-analysis were more concentrated at the top of the funnel plot and evenly distributed on both sides of the mean effect size. In addition, the results of Begg’s rank correlation test indicated that *p* = 0.74 > 0.01. Therefore, the original hypothesis was accepted, indicating that the funnel plot is symmetric. Figure 3 demonstrates a symmetric funnel plot, indicating minimal publication bias in the included studies.

Also, we used the fail-safe *N* and Egger’s regression coefficient to conduct the quantitative test for publication bias. Specifically, the fail-safe *N* indicates how many missing effect sizes are required to bring an effect size to a nonzero value, and the value of the fail-safe *N* at the *p* level of 0.05 should be compared with 5*K* + 10, where the study number is *K* and the sample size is *N*. When it is less than 5*K* + 10, it means there is a serious publication bias problem; on the contrary, the larger the fail-safe *N* is, the less likely there is a publication bias problem [70]. The result showed that the fail-safe *N* for the relationship between interaction and value co-creation in OCIC is 4892 at the *p*-value of 0.05, which is far greater than the critical value of 5*K* + 10 (i.e., 335; *K* is the study number), indicating that at least 4892 unpublished studies would need to be included to reduce the combined effect of the study to a non-significant level [71]. Furthermore, Egger’s regression results showed that *β* = 1.03, *t* = 0.43, 95% CI [−3.74, 5.81] and *p* = 0.67 > 0.05, suggesting that the missing studies do not lead to an underestimation of the effect of the relationship between interaction and value co-creation, that is, publication bias does not affect the main findings of the meta-analysis [72]. Finally, the results of the trim-and-fill method found that the correlation coefficient was significant (*r* = 0.495, 95% CI [0.446, 0.540]) after adding nine studies to the right side. The corrected effect size was slightly higher than the effect size before correction (*r* = 0.453), but the difference was only 0.042. In addition, adding studies to the left side was not necessary.

In sum, all of the above results indicate that there is no serious publication bias problem in the present study, and the effect sizes have high stability.

### 4.3. Main Effect Analysis

A random-effects model was used to test the strength of the correlation between interaction and value co-creation in OCICs. The results showed that the overall correlation coefficient (*r*) between the two variables was 0.453, with a 95% confidence interval of [0.405, 0.499], excluding 0. Concerning the explanatory criteria for the magnitude of the correlation coefficient proposed by Gignac and Szodorai (2016), 0.10, 0.20, and 0.30 represent low, moderate, and strong correlation [73]. It can be considered that there is a strong positive correlation between interaction and value co-creation. The sensitivity analysis revealed that after excluding any one sample, the effect size r fluctuated between 0.444 and 0.459 and the difference from the main effect value of 0.453 is not significant, which indicates the results are robust. Hypothesis 1 predicted a significant positive effect of interaction on value co-creation. The results confirmed this with an effect size of *r* = 0.453 (*p* < 0.001), indicating a strong positive relationship. Therefore, the results support Hypothesis 1.

### 4.4. Moderator Analyses

We used subgroup analysis to test whether moderators had an effect on the relationship between interaction and value co-creation. The results are presented in Table 5, which shows the moderating effects of the types of interaction, the types of OCICs, the number of involved OCICs, and the cultural background on the interaction and value co-creation relationship, which were examined separately using a random effects model. We discuss the results of the heterogeneity tests for each moderator separately in the following section.

Types of interaction: The correlation between interaction and value co-creation was significant when the types of interaction differed. The results of the subgroup analysis (65 effect values) showed that the *Q* value (between groups) was 11.515, with *p* = 0.021 < 0.05. The effect size estimates involving both types of HHI (i.e., user–user interaction and user–firm agent interaction) were the strongest (*r* = 0.590, *p* < 0.001), followed by both user–user interaction and HCI (r = 0.510, *p* < 0.001) and HCI (*r* = 0.415, *p* < 0.001), user–user interaction (*r* = 0.414, *p* < 0.001), user–firm agent interaction (*r* = 0.382, *p* < 0.001). These results suggest that interaction can significantly enhance value co-creation when two types of interactions are involved at the same time; furthermore, HCI and user–user interaction have little difference in their effects on the relationship between variables, while user–firm agent interaction has the weakest effect. Hypothesis 2 predicted that interaction types significantly moderate the relationship between interaction and value co-creation, and user–user interactions have stronger effects compared to user–firm agent interactions and HCI. The results partially confirmed this with *Q* = 11.515 (*p* < 0.05) and the effect size of HCI (*r* = 0.451, *p* < 0.001) was the strongest compared with user–user interaction and user–firm interaction. Therefore, Hypothesis 2 was partially supported. Further, these findings suggest that combining human–human and human–computer interactions can maximize value co-creation, offering insights for designing interactive platforms.

Types of online collaborative innovation communities: The relationship between interaction and value co-creation was significantly different when the types of OCICs were different (*QB* = 14.892, *p* = 0.005 < 0.01). The estimated effect size of brand communities embedded in social media (*r* = 0.551, *p* < 0.001) was significantly higher than interest-based communities (*r* = 0.469, *p* < 0.001), interest-based communities were significantly higher than relational communities (*r* = 0.382, *p* < 0.001), relational communities were weakly higher than generalized transaction-based communities (*r* = 0.371, *p* < 0.001) with negligible higher values, and generalized transaction-based communities were significantly higher than crowdsourcing communities (*r* = 0.272, *p* < 0.01). The 95% CI under the five OCIC types does not contain 0. Hypothesis 3 predicted that the OCIC types significantly moderate the relationship between interaction and value co-creation and brand communities embedded in social media have the most significant effect, followed by relational OCs and interest-based OCs, while crowdsourcing communities have the weakest effect. The results partially confirmed this with *Q* = 14.892 (*p* < 0.01), and the effect size of *r* = 0.551 (*p* < 0.001) of brand communities embedded in social media was the strongest, but interest-based communities were significantly higher than relational communities (*r* = 0.382, *p* < 0.001). Thus, Hypothesis 3 was partially confirmed. Practically, the strong effect of brand communities embedded in social media (*r* = 0.551, *p* < 0.001) and interest-based OCs (*r* = 0.469, *p* < 0.001) underscores that organizations can leverage social media as well as inspire greater user interest to promote value co-creation that generates additional benefits for the organization.

Number of involved online collaborative innovation communities: The moderating effect of the number of OCICs involved in the study on the relationship between interaction and value co-creation was significant. The results of the subgroup analysis (65 effect sizes) showed that the *Q* value (between groups) was 6.533, with *p* = 0.011 < 0.05. The correlation coefficient between interaction and value co-creation was higher when multiple OCICs were included in the examination (*r* = 0.490, *p* < 0.001) compared to the study involving only one single OCIC (*r* = 0.375, *p* < 0.001), and the 95% CI did not contain 0 in either case. Hypothesis 4 predicted that the number of involved OCICs moderates the relationship between interaction and value co-creation and the positive correlation between interaction and value co-creation is stronger in a study that includes multiple OCICs. The results confirmed this with *Q* = 6.533 (*p* < 0.05); the correlation coefficient *r* = 0.49 (*p* < 0.001) between interaction and value co-creation was higher when multiple OCICs were included. Therefore, Hypothesis 4 was supported. Furthermore, online community managers can consider expanding the number of OCs accessible to users to promote value co-creation by increasing the number of OCICs that can link to other OCICs.

Cultural background: The moderating effect of the participant’s cultural background on the relationship between interaction and value co-creation was not significant. The results of the subgroup analysis (51 effect sizes) revealed that the *Q* value (between groups) was 0.237, with *p* = 0.627 > 0.05. The results were not significant. Hypothesis 5 predicted that the cultural background moderates the relationship between interaction and value co-creation. The results not confirmed this with *Q* = 0.237 (*p* > 0.05) and an effect size of *r* = 0.453 (*p* < 0.001), indicating the moderating effect was not significant. Thus, Hypothesis 5 was not supported.

## 5. Discussion

Based on the service ecosystem framework, this study used a meta-analysis approach to quantitatively review 65 empirical studies from 63 research articles examining the relationship between interaction and user value co-creation in the OCICs, which included a total of 25,185 participants. The results of the main effects of the meta-analysis revealed a strong positive correlation relationship between interaction and value co-creation (*r* = 0.453, *p* < 0.001), thus confirming Hypothesis 1, which predicted a positive correlation between interaction and value co-creation. Our findings align with prior conclusions indicating that interaction significantly promotes more user value co-creation behaviors [74,75], contradicting studies that found either a negative or even non-significant relationship between actors’ interaction and value co-creation [16,17]. Additionally, we examined the potential moderating effects of types of actors’ interaction, types of OCICs, the number of involved OCICs, and the user’s cultural background at the micro, meso, and macro levels of the service ecosystem framework, offering a more nuanced understanding of how these factors shape the interaction–value co-creation dynamics. Through the construction of a stable and well-organized online collaborative innovation community service ecosystem, exploring an ideal “all win more” model that can guide all parties to participate in value co-creation will contribute to the new development of the value co-creation paradigm.

### 5.1. The Relationship Between Interaction and Value Co-Creation in Online Collaborative Innovation Communities

Our main effect findings support and shed light on activities occurring within micro-contexts of the service ecosystem framework [13,30], where dyadic interactions among multiple actors facilitate value co-creation in OCICs by eliciting the exchange and integration of operant resources, thereby answering Q1. It is consistent with previous research concluding that actor interaction is the most significant factor promoting various forms of user value co-creation in OCICs (e.g., knowledge co-creation [6]; creative idea generation [55]). However, contrary to predictions from the attention-based view based on resource scarcity, our results indicate that an increase in interactions within OCICs does not diminish user engagement in value co-creation. This outcome may be attributed to users’ motivations in OCICs, which often include a desire for general entertainment rather than strictly instrumental goals [76]. The frequent interactions within OCICs may thus serve to engage users further in reciprocal value co-creation by fulfilling their emotional and hedonic needs, even amid the cognitive loads imposed by processing informational content across different domains.

### 5.2. Moderators That Impact the Relationship Between Interaction and Value Co-Creation in Online Collaborative Innovation Communities

We answered Q2 by further analyzing the moderators affecting the relationship between interaction and value co-creation at the micro, meso, and macro levels of the service ecosystem, respectively.

First, at the micro level, our findings suggest that the moderating effect of the different types of actor-to-actor interaction on the relationship between interaction and value co-creation is significant (*Q* = 11.515, *p* < 0.05.), thus confirming Hypothesis 2. Consistent with our hypothesis, the more types of interactions involved in the study, the stronger the correlation between the interaction and value co-creation. Any interaction in the OCICs will result in the acquisition of heterogeneous knowledge that is distinct from their knowledge repositories; with the increase in interaction types, the user’s knowledge reserves expand, making it easier for them to contribute more new ideas [55]. For studies that only involved one type of interaction, the difference in effect size between HCI and user–user interaction was negligible. As both represent voluntary and proactive interaction choices of users in OCICs [41], the intensity of internal motivations that may drive users to engage in value co-creation is similar. In addition, our results found the moderating role of dyadic interactions between human and nonhuman actors (i.e., HCI). It implies that communication technology and IT-mediated interactive platforms provide effective channels for users’ operant resource flows from the technological level to support users’ value co-creation through resource exchange and integration [77]. However, user–firm agent interaction has the lowest effect size. This is due to firm representatives of the company being given privileged power to manage the OCICs by selectively interacting with innovators who provide high-quality knowledge while controlling the platform’s discourse [78]. Such top-down interactions that serve the business may reduce users’ trust.

The results of our meta-analysis demonstrate the moderating role of different types of OCICs as mediated actors in the meso level of service ecosystems (*Q* = 14.892, *p* < 0.01); therefore, Hypothesis 3 was supported. OCICs provide a virtual interface for actor-to-actor interactions [9] to afford new potentialities for value co-creation. In particular, first, the relationship between interaction and value co-creation was the strongest among brand communities embedded in social media. The triple interactive mangle advantage of the unique advantages of social media platforms, the vigorous promotion by firms, and the passion of users for brands have contributed to the fact that interaction in brand communities embedded in social media triggers a stronger sense of belonging and community identity among users [21]. Second, our results found stronger effect sizes for interest-based OCs than online relational communities, which is inconsistent with our hypothesis. As compared to relational communities such as online health communities, which have higher member churn rates [79], user interactions in interest-based online communities are built on the premise that they are interested in or passionate about the domain-specific content, peer recognition and personal achievement realized through interactions, which motivate users to engage in more value co-creation activities [80]. While generalized transaction-based and crowdsourcing online communities are business-driven and aim to gather more creative input from external users to guide business decisions [81], the user’s active and purely voluntary participatory role is hard to represent. Further, users in crowdsourcing communities tend to engage in one-time virtual idea competitions for prizes or industry reputation, where the more frequent disruptive behaviors among members lower users’ efforts [50].

Furthermore, Hypothesis 4 predicted that the number of interactive platforms involved at the meso level also affects the relationship between interaction and value co-creation, which was supported by our findings (*Q* = 6.533, *p* < 0.05). That is, the positive effect of interaction on value co-creation was stronger when the number of OCICs included in the study was two or more. It is related to the possibility of increasing access to the heterogeneity of knowledge and the diversity and quality of ideas possessed by users through the expansion of network size [82] and channel capacity [83].

We attempted to analyze the effects of users’ cultural backgrounds on activities in OCICs at a broader macro level to examine Hypothesis 5. However, there is no evidence in our results for the moderating effect of both Eastern and Western cultural backgrounds on interaction and value co-creation in OCICs (*Q* = 0.237, *p* > 0.05). The lack of significant cultural background moderation may reflect the increasing globalization of digital communities, where cultural differences have less impact due to shared online norms. Moreover, with the changing times and the emergence of global cultural integration and multicultural coexistence, socio-cultural values worldwide are generally consistent in showing an evolving tendency of rising individualism and declining collectivism [84]. Thus, interaction behavior in OCICs is less affected by the varying degrees of trust in in-circle and out-of-circle groups that accompany cultural differences. These factors may have caused the insignificant moderating effect of cultural background on interaction and value co-creation relationships within OCICs.

**Figure 4:**
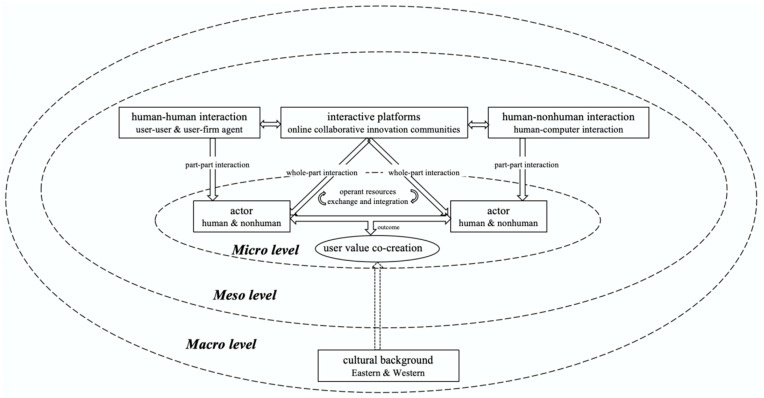
OCIC-based service ecosystem.

Overall, as shown in Figure 4, combining the service ecosystem framework and the results of the meta-analysis, we constructed the OCIC-based service ecosystem. The system presents the impact of different types of interactions, the diversity of OCICs, and the users’ cultural background on the micro foundation activities (i.e., the process of exchanging and integrating operant resources through interactions to achieve value co-creation) at the micro, meso, and macro levels, revealing that value co-creation in OCICs is a phenomenon of internal and external environment interplay that occurs within reciprocal multi-actor interactions and is mediated by interactive platforms using digital technologies to provide actor networks. In addition, combining the results illustrated in Figure 4 reveals that there are mutual interactions between the different levels within the OCIC-based service ecosystem, and this includes both part–part interactions and whole–part interactions. Specifically, the interplay between micro-level user interactions and meso-level online community structures suggests that platform design must facilitate seamless transitions between individual and group engagements, while optimizing the interface of interactive platforms with the characteristics of interactions between different actors to create a better interactive experience for users. Although the interplay between macro-level cultural backgrounds and micro-level actors’ interactions is not significant, it provides further evidence that due to the trend of cultural convergence and globalization as well as the fact that online communities are not limited by space, more and more users from different cultural backgrounds in the East and West will interact through community platforms, so the access and design of the platforms should be more diversification and more accessible.

### 5.3. Theoretical and Practical Implications

Our research has two theoretical implications. First, while service marketing scholars and practitioners have long recognized the decisive effect of actor-to-actor interactions on value co-creation [4,8], some studies have found that interaction does not always mean value co-creation [20]; there has been no consensus up to this point on this relationship among the extant empirical studies. By integrating existing research and reconciling previous conflicting findings, this study contributes to the debate on the effect of actors’ interactions on value co-creation in virtual communities by using meta-analysis to provide cumulative insights and a stronger conclusion regarding the overall positive effect between interaction and user value co-creation than those found in traditional empirical studies. Second, with the convergence of the physical and digital world in a mixed reality, we dynamically explore a host of potential moderating factors according to the service ecosystem framework at the micro, meso, and macro levels, respectively, to reveal the roles of different types of actor-to-actor interactions, types of OCICs, and the more complex cultural backgrounds in the interactions and value co-creation. Complementing previous theoretical and conceptual research, this study empirically explores the relationship between the different layers of ecosystems supported by interactive digital platforms for the first time, going beyond the traditional S-D logic, and we respond to the call for service ecosystems as a unit of analysis for value co-creation [13,85] to better understand the relationship between multi-actor interaction and value co-creation.

This study also yields several managerial implications for value co-creation activities in OCICs. First, the present results found a significant positive correlation between interaction and user value co-creation in OCICs. It implies that rather than being unilaterally determined, value in OCICs may be co-created through social interactions among all human stakeholders and nonhuman actors involved in the OCIC ecosystem. Therefore, managers should create diverse forms of actor interactions and dialogues in OCIC environments to promote interdependence and transformation of actors’ operant resources and enhance OCICs’ dynamics and influence by contributing and creating more value together. Moreover, the present study also found that correlation strength was moderated by the types of interaction, which reveals that community managers should create opportunities that encompass different types of interactions. Not only do community managers need to promote user interactions by setting top trending posts, they also need to update community information, post salient topics, and reward active users. Since interactive platforms as nonhuman actors can catalyze user interactions [86], OCICs’ developers should improve the platform’s personalized interactive functions (e.g., search and page navigation functions) under the characteristics of different users’ browsing hours, usage habits, and preferred boards, to enhance the platform’s ease-of-use and user-friendliness in HCI. Further, platform developers learn users’ computer-mediated interaction behaviors such as browsing and information retrieval within OCICs through real-time monitoring in the backend, so as to provide diversified strategic guidance for the optimization of the interface of the platform (e.g., information and graphics presentation, page navigation and design, link use and predictive systems) [41]. Furthermore, our study found that the types of OCICs influence the strength of the relationship between interaction and value co-creation, which indicates that differentiated policies should be implemented across different OC platforms to promote interaction among actors. In particular, for business-sponsored online communities, companies may consider embedding online communities in social media to capture more useful and creative ideas from loyal users for further consideration and implementation. Finally, since differences in the number of involved OCICs also affect user behavior, researchers need to consider including multiple OCICs simultaneously in future studies to ensure the quality of the data and the generalizability of the findings.

### 5.4. Limitations and Future Research Directions

There are still some limitations in this study. First, although this study considered the moderating effects of multiple factors as thoroughly as possible, it is still not comprehensive and complete. We did not consider other participant characteristics (e.g., gender, age, occupation) in the meta-analysis due to many studies that were unable to capture these details. However, studies have reported the critical role of age in user engagement in OCs, with younger users being more willing to acquire and share information through online communities [87], while older users over the age of 60 are less likely to participate in value co-creation in OCs as passive users [88]. Hence, subsequent meta-analysis research should consider the influence of other potential demographic moderating variables such as age. Researchers could use data crawling to obtain the basic personal registration information made public by the users, and further segment the users’ information such as gender and age to gain insights into the impact of this information on the relationship between interaction and user value co-creation. Second, due to the absence of standardized conceptual definitions and measurements, users’ value co-creation in OCICs covers a wide range of behaviors (e.g., information sharing, participation and citizenship behaviors, crowdsourcing, and creative idea generation). This might ignore the differences in the definition of constructs across studies. Thus, future research could explore the interaction’s role in the context of more segmented user behaviors. Meanwhile, to further consider whether interactions in OCICs have divergent effects on co-creation behavior at different levels, future research could focus on the shallow (e.g., searching, liking) and deep (sharing information, creating new ideas) levels of value co-creation, respectively [5]. Finally, the studies included in our meta-analysis were mainly correlational or cross-sectional studies, and the meta-analysis’s findings could only reveal the existence of a linear correlation between interaction and value co-creation. In the future, experimental studies, longitudinal tracing studies, or the use of randomized instrumental variables in the form of questionnaires should be included to further uncover the causal relationship between interaction and value co-creation [89].

## 6. Conclusions

We clarified the relationship between interaction and user value co-creation in online collaborative innovation communities and its influencing factors using meta-analysis, which provides relevant additions to the contradictory findings of the existing studies, and also guides the subsequent empirical studies in the field of online communities. We draw the following conclusions: (1) interaction has a significantly positive impact on user value co-creation within OCICs. (2) Interaction types (human–human and human–computer interaction) moderate the relationship between interaction and value co-creation. (3) The types of OCICs (business-sponsored or socially constructed online communities) play a significant moderating role in interaction and value co-creation relationships. (4) The number of involved OCICs (one or multiple OCICs) played a significant moderating role in the relationship between interaction and value co-creation. (5) The moderating effect of users’ cultural background (individualism or collectivism) in OCICs was not significant.

## Figures and Tables

**Table 1:** A description of types of OCICs included in our meta-analysis.

General Types of OCICs	Description	Operationalized Definition of Specific Types of OCICs	Study Number
Business-sponsored OCs	Serving the interests of firms	Generalized transaction-based online communities: these communities usually have their own specialized websites as a platform to liaise with external users and are characterized by their commercial nature E.g., Starbucks’ MyStarbucksIdea, Dell’s IdeaStorm	18
		Crowdsourcing communities: these communities leverage the crowd wisdom and collective intelligence of an online community to serve specific organizational goals E.g., one-time contests	5
		Brand communities embedded in social media: these communities act as an important mediator for companies to connect the OC triangle relationship E.g., brand community embeded in Facebook	21
Socially constructed OCs	Peer-to-peer communities, users are motivated to join the OC to seek information on topics of interest	Interest-based communities: these consist of a dispersed collection of people who share common interests and expertise in a given topic E.g., online Q&A community	18
		Relational communities: these communities consist of people with similar life experiences gathering together and building meaningful relationships through shared experiences E.g., online health communities	3

**Table 2:** Summary of research hypotheses.

Hypothesis	Specific Research Hypothesis	Corresponding Service Ecosystem Framework Levels
Hypothesis 1	Interaction between actors in OCICs has a significant positive effect on user value co-creation behavior.	Micro level of service ecosystems
Hypothesis 2	The types of interaction among actors significantly moderate the relationship between interaction and user value co-creation. Specifically, user–user interactions have stronger effects compared to user–firm agent interactions and HCI. In addition, the greater the number of interaction types involved in OCICs, the stronger the correlation between interaction and value co-creation.	Micro level of service ecosystems
Hypothesis 3	The types of online collaborative innovation communities significantly moderate the relationship between interaction and value co-creation. Specifically, brand communities embedded in social media have the most significant effect, followed by two types of socially constructed online communities, where online relational communities have a relatively stronger effect and crowdsourcing communities have the weakest effect when compared to other types of OCICs.	Meso level of service ecosystems
Hypothesis 4	The number of involved OCICs moderates the relationship between interaction and value co-creation. Specifically, there will be a stronger positive correlation between interaction and value co-creation in a study that includes multiple OCICs together compared to a study that includes only one OCIC.	Meso level of service ecosystems
Hypothesis 5	The cultural background of OCIC users moderates the relationship between interaction and value co-creation. The positive impact of interaction on value co-creation is more pronounced among users from individualist Western cultures than those from collectivist Eastern cultures.	Macro level of service ecosystems

**Table 3:** A description of moderators included in our meta-analysis.

Moderators	Operationalized Definition of Specific Moderators	Selection Criteria and Importance to the Study
Types of interaction	Interactions occurring between multiple actors in OCICs, comprising both human–human interaction (e.g., user–user and user–firm agent interaction) and human–computer interaction.	As value is co-created by multiple actors, it is necessary to examine the impact of different types of actor’s interactions on the value co-creation within OCICs.
Types of online collaborative innovation communities	It refers to OCs created for different purposes. Specifically, OCICs oriented towards business innovation can be categorized into three types: generalized transaction-based communities, crowdsourcing communities, and brand communities embedded in social media. Two socially constructed OCs dedicated to interest and information exchange are also classified as OCICs, including interest-based and relational OCs.	As different types of digitalized interactive platforms, different OCICs are distinct and active mediators that provide unique virtual interfaces for actors’ value co-creation.
Number of involved online collaborative innovation communities	It refers to whether single or multiple OCICs are included as data collection platforms in empirical studies.	Through the configuration of multiple related interactive platforms, an “all win more” approach to value co-creation becomes achievable. Thus, actors’ interactions across a variety of interactive platforms will influence users’ value co-creation experiences.
Cultural background	It refers to the different cultural backgrounds (i.e., collectivism and individualism) of users within OCICs.	Users from different cultures have different preferred interaction styles, which can affect their value co-creation behavior.

**Table 4:** Test of heterogeneity with *Q*-value, *I*^2^ and *τ*^2^.

	Heterogeneity				*τ*²			
Model	*Q*-Value	*df*(*Q*)	*p*-Value	*I* ^2^	*τ* ^2^	Standard Error	Variance	*τ*
Random	1409.29	64	0.000	95.459	0.055	0.011	0.000	0.234

**Table 5:** Results of the subgroup analyses.

Moderator	*K*	*N*	Effect Size and 95% CI		Test of Null (2-Tail)		Heterogeneity Test	
			Point Estimate	95% CI	Z-Value	*p*-Value	*QB* (df)	*p*-Value
Types of interaction							11.515 (4) *	0.021
User–user, HCI	8	3587	0.510	[0.440, 0.573]	12.268	0.000		
User–user, user–firm agent	10	3876	0.590	[0.457, 0.698]	7.193	0.000		
HCI	4	1774	0.415	[0.376, 0.453]	18.544	0.000		
User–user	37	14,580	0.414	[0.346, 0.477]	10.967	0.000		
User–firm agent	6	1368	0.382	[0.235, 0.511]	4.860	0.000		
Types of OCICs							14.892 (4) **	0.005
Brand communities embedded in social media	21	8475	0.551	[0.458, 0.633]	9.652	0.000		
Interest-based	18	6520	0.469	[0.406, 0.528]	12.714	0.000		
Relational	3	1611	0.382	[0.188, 0.546]	3.726	0.000		
Generalized transaction-based	18	7041	0.371	[0.306, 0.433]	10.425	0.000		
Crowdsourcing	5	1538	0.272	[0.086, 0.440]	2.836	0.005		
Number of involved OCICs							7.343 (1) **	0.007
One	22	7267	0.375	[0.305, 0.441]	9.799	0.000		
Multiple	43	17,918	0.490	[0.431, 0.544]	14.146	0.000		
Cultural background							0.237 (1)	0.627
Eastern	34	13,058	0.469	[0.418, 0.517]	15.726	0.000		
Western	17	7249	0.435	[0.298, 0.555]	5.744	0.000		

Note: *QB*, heterogeneity between groups; *N*, cumulative sample size across all studies; *K*, number of effect values; CI, confidence interval. ** *p* < 0.01; * *p* < 0.05.

## Data Availability

All data included in this study are available upon request by contact with the first author Chunzhen Wang (wangchunzhen@mails.ccnu.edu.cn).

## Supplementary Materials

The following supporting information can be downloaded at: https://www.mdpi.com/article/10.3390/bs14121177/s1, Table S1: Summary and basic information of studies included in the meta-analysis.
