# Supplementary material for: A Meta-Analysis of the Effects of Interaction on Value Co-Creation in Online Collaborative Innovation Communities Based on the Service Ecosystem Framework
Source: Behav Sci (Basel). 2024 Dec 9;14(12):1177. doi: 10.3390/bs14121177 (PMC11673022; doi:10.3390/bs14121177)
Supplement: Supplementary file 1 [file behavsci-14-01177-s001.zip › behavsci-3312810-supplementary.pdf]

Supplementary Table S1: Summary and basic information of studies included in the meta-analysis.

Authors, Year	Sample Size	Effect Size (r)	Types of Interaction	Types of Online Communities	Number of Online Communities	Cultural Background	Female Ratio	Publication Type
Barney-McNamara (2021)	255	0.685	User–user	Brand communities embedded in social media	Multiple	Western	0.412	Dissertation
Braun et al. (2016)	255	0.11	User–firm agent	Generalized transaction-based	One	Western	0.46	Published
Bruhn et al. (2013)	330	0.55	User–firm agent	Generalized transaction-based	Multiple	—	0.052	Published
Bui and Jeng (2022)	711	0.39	User–user	Relational	Multiple	Eastern	0.453	Published
Chen et al. (2020)	338	0.325	User–user	Interest-based	One	Eastern	0.825	Published
Chen et al. (2021)	477	0.39	HCI	Interest-based	Multiple	Eastern	0.636	Published
Cheung et al. (2021)	315	0.522	User–user, user–firm agent	Brand communities embedded in social media	Multiple	Eastern	0.695	Published
Chiang et al. (2017)	373	0.716	User–user	Brand communities embedded in social media	Multiple	—	0.38	Published
Cho et al. (2014)	63	0.191	User–user	Interest-based	One	Eastern	0.381	Published
Claffey and Brady (2019)	307	0.275	User–user	Generalized transaction-based	One	—	0.382	Published
Cornelissen et al. (2019)	104	0.539	User–user, user–firm agent	Interest-based	Multiple	—	—	Published
Cui et al. (2022)	387	0.454	HCI	Generalized transaction-based	Multiple	Eastern	0.628	Published
Dai et al. (2019)	415	0.42	User–user	Brand communities embedded in social media	Multiple	—	0.528	Published
de Silva (2021)	374	0.66	User–user	Brand communities embedded in social media	Multiple	Eastern	0.473	Published
Diffley and McCole (2015)	120	0.086	User–user	Generalized transaction-based	Multiple	Western	—	Published
Ebrahimi et al. (2021)	433	0.4	User–user	Interest-based	Multiple	Western	0.379	Published
Fang et al. (2021)	591	0.51	User–user, HCI	Brand communities embedded in social media	Multiple	—	0.494	Published
Guan et al. (2022)	593	0.639	User–user	Interest-based	Multiple	Eastern	0.481	Published
Hsu (2017)	646	0.575	User–user, user–firm agent	Generalized transaction-based	One	Eastern	0.554	Published
Huang et al. (2013)	284	0.42	User–user	Interest-based	One	Eastern	0.546	Published
Huang et al. (2020)	569	0.37	User–user	Generalized transaction-based	Multiple	Eastern	0.533	Published
Huang et al. (2022)	332	0.62	User–user, HCI	Brand communities embedded in social media	One	Eastern	0.364	Published
Hussain et al. (2022)	286	0.488	User–user, HCI	Interest-based	Multiple	Eastern	0.573	Published
Kamboj et al. (2018)	407	0.25	User–user	Brand communities embedded in social media	Multiple	Eastern	0.41	Published
Khan (2014)	757	0.125	User–user	Brand communities embedded in social media	Multiple	Western	0.705	Dissertation
Kim and Kim (2021)	376	0.619	User–user	Interest-based	Multiple	Eastern	0.524	Published
Ko et al. (2019)	168	0.292	User–user	Generalized transaction-based	One	Eastern	—	Published
Latif et al. (2022)	608	0.196	User–user	Relational	Multiple	Eastern	—	Published
Lee and Kim (2022)	513	0.638	User–user, HCI	Interest-based	Multiple	Western	0.532	Published
Li et al. (2016)	381	0.04	User–user	Crowdsourcing	Multiple	—	—	Published

Liang et al. (2020)	63	0.549	User–agent	Interest-based	One	Eastern	0.481	Conference paper
Liu (2016)	591	0.071	User–user	Crowdsourcing	One	Western	—	Conference paper
Liu et al. (2014)	403	0.35	User–user	Generalized transaction-based	One	Eastern	0.474	Published
Liu et al. (2019)	292	0.541	User–user	Relational	One	Eastern	0.538	Published
Luo and Li (2022)	485	0.344	User–user, HCI	Generalized transaction-based	Multiple	Eastern	0.336	Published
Luo et al. (2019)	712	0.257	User–user	Interest-based	One	Eastern	0.441	Published
Nadeem et al. (2021)	485	0.839	User–user, user–firm agent	Brand communities embedded in social media	Multiple	Western	0.659	Published
Nambisan and Baron (2009)	152	0.23	User–user	Generalized transaction-based	Multiple	—	—	Published
Naqvi et al. (2021)	320	0.79	User–user, user–firm agent	Brand communities embedded in social media	Multiple	Eastern	0.469	Published
Naumann et al. (2020)	625	0.373	User–user, user–firm agent	Interest-based	Multiple	Western	0.525	Published
Onofrei et al. (2022)	403	0.226	User–user	Brand communities embedded in social media	Multiple	Western	0.268	Published
Pham et al. (2020)	248	0.489	User–user	Brand communities embedded in social media	Multiple	Eastern	0.29	Published
Rautela et al. (2021)	213	0.522	User–user	Interest-based	Multiple	Eastern	0.437	Published
Reitz (2012)	233	0.455	User–user, HCI	Brand communities embedded in social media	Multiple	—	0.69	Dissertation
Rubio et al. (2019)	600	0.503	User–user, user–firm agent	Interest-based	Multiple	Western	0.503	Published
Samala and Katkam (2020)	466	0.562	User–user	Brand communities embedded in social media	Multiple	—	0.46	Published
Sanz-Blas et al. (2017)	642	0.683	User–user	Brand communities embedded in social media	Multiple	Western	0.51	Published
Sanz-Blas et al. (2019)	370	0.45	User–user	Brand communities embedded in social media	Multiple	—	0.51	Published
Seeber et al. (2017)	149	0.317	User–user	Crowdsourcing	One	—	—	Conference paper
Shao and Ross (2015)	450	0.354	User–user, user–firm agent	Brand communities embedded in social media	Multiple	Western	0.67	Published
Shin et al. (2020)	240	0.342	User–firm agent	Generalized transaction-based	One	Western	0.529	Published
Shin et al. (2020)	240	0.282	User–firm agent	Generalized transaction-based	One	Western	0.517	Published
Shin et al. (2020)	240	0.436	User–firm agent	Generalized transaction-based	One	Western	0.509	Published
Suh et al. (2004)	198	0.477	User–user	Interest-based	Multiple	Eastern	0.611	Published
Ul Islam and Rahman (2017)	430	0.46	User-user, HCI	Brand communities embedded in social media	Multiple	Eastern	0.43	Published
Utami et al. (2022)	717	0.515	User–user, HCI	Generalized transaction-based	Multiple	Eastern	0.554	Published
Vohra and Bhardwaj (2019)	209	0.586	User–user, user–firm agent	Brand communities embedded in social media	Multiple	Eastern	0.2	Published
Wairimu (2020)	122	0.583	User–user, user–firm agent	Crowdsourcing	One	—	—	Dissertation
Wang et al. (2020)	400	0.65	User–user	Brand communities embedded in social media	Multiple	Western	0.636	Published
Wen et al. (2022)	528	0.396	HCI	Generalized transaction-based	Multiple	Eastern	0.29	Published

Weng et al. (2019)	295	0.33	User–user	Crowdsourcing	One	Eastern	0.427	Conference paper
Yang and Li (2016)	885	0.271	User–user	Generalized transaction-based	One	—	—	Published
Yang et al. (2021)	369	0.555	User–user	Generalized transaction-based	Multiple	Eastern	0.276	Published
Zhang et al. (2017)	260	0.5	User–user	Interest-based	One	Eastern	0.554	Published
Zhang et al. (2019)	382	0.432	HCI	Interest-based	One	Eastern	0.45	Published

Note: Among the types of interactions, there are two major categories, i.e., human–human interaction and human–computer interaction (HCI), with user–user interaction and user–firm agent interaction classified as human–human interaction.
